# Spatiotemporal Analyses of 2 Co-Circulating SARS-CoV-2 Variants, New York State, USA

**DOI:** 10.3201/eid2803.211972

**Published:** 2022-03

**Authors:** Alexis Russell, Collin O’Connor, Erica Lasek-Nesselquist, Jonathan Plitnick, John P. Kelly, Daryl M. Lamson, Kirsten St. George

**Affiliations:** New York State Department of Health, Albany, New York, USA (A. Russell, C. O’Connor, E. Lasek-Nesselquist, J. Plitnick, J.P. Kelly, K. St. George);; State University of New York at Buffalo, Buffalo, New York, USA (C. O’Connor);; State University of New York at Albany, Albany (E. Lasek-Nesselquist, K. St. George)

**Keywords:** COVID-19, coronavirus disease, SARS-CoV-2, severe acute respiratory syndrome coronavirus 2, viruses, respiratory infections, zoonoses, vaccine-preventable diseases, spatiotemporal analyses, New York, United States

## Abstract

The emergence of novel severe acute respiratory syndrome coronavirus 2 (SARS-CoV-2) variants in late 2020 and early 2021 raised alarm worldwide because of their potential for increased transmissibility and immune evasion. Elucidating the evolutionary and epidemiologic dynamics among novel SARS-CoV-2 variants is essential for understanding the trajectory of the coronavirus disease pandemic. We describe the interplay between B.1.1.7 (Alpha) and B.1.526 (Iota) variants in New York State, USA, during December 2020–April 2021 through phylogeographic analyses, space-time scan statistics, and cartographic visualization. Our results indicate that B.1.526 probably evolved in New York City, where it was displaced as the dominant lineage by B.1.1.7 months after its initial appearance. In contrast, B.1.1.7 became dominant earlier in regions with fewer B.1.526 infections. These results suggest that B.1.526 might have delayed the initial spread of B.1.1.7 in New York City. Our combined spatiotemporal methodologies can help disentangle the complexities of shifting SARS-CoV-2 variant landscapes.

The emergence of a novel severe acute respiratory syndrome coronavirus 2 (SARS-CoV-2) variant B.1.1.7 (Alpha) in the United Kingdom in late 2020 raised alarm worldwide and prompted major reassessment of the management, surveillance, and projected future of coronavirus disease (COVID-19) (*1*,*2*). Evidence of increased transmissibility and potential immune evasion prompted the World Health Organization to designate B.1.1.7 a variant of concern (VOC) in December 2020 (*3*–*5*; W.A. Haynes et al., unpub. data, https://doi.org/10.1101/2021.01.06.20248960). The emergence of B.1.1.7 and additional novel SARS-CoV-2 variants with competitive advantages has resulted in the localized dominance of single variants (E. Volz et al., unpub. data, https://doi.org/10.1101/2020.12.30.20249034) and raised concern for increases in COVID-19 incidence (*6*).

Novel variant B.1.526 (Iota) arose within New York State (NYS), USA, in late 2020 (E. Lasek-Nesselquist et al., unpub. data, https://doi.org/10.1101/2021.02.26.21251868) (*7*) and quickly increased in proportion throughout the state, leading to a noticeable shift in lineage distribution during early 2021 (E. Lasek-Nesselquist et al., unpub. data, https://doi.org/10.1101/2021.02.26.21251868) (*31*). The World Health Organization designated B.1.526 as a variant of interest (VOI) because of its increase in prevalence coupled with mutations associated with immune evasion (*8*). Despite these concerns, an epidemiologic assessment of B.1.526 in NYC during January–April 2021 found that the lineage did not cause more severe disease and was not associated with increased risk for reinfection or vaccine breakthrough (*9*). However, an epidemiologic study of NYS during late 2020–May 2021 concluded that B.1.526 was 35% more transmissible than non-VOC and non-VOI lineages circulating at the time (*10*).

Genomic surveillance of COVID-19 is a crucial tool to monitor and assess the physiologic and epidemiologic characteristics of SARS-CoV-2 variants as they emerge. The New York State Department of Health (NYSDOH) substantially expanded its genomic surveillance program in December 2020, with the aim of sequencing a more representative subset of COVID-19 cases across the state to track the spread and impact of novel variants. A robust genomic surveillance system enables assessment of changes in variant distribution over precise temporal and spatial scales.

This study employed spatial scan statistics paired with phylogeographic analyses to describe the shifting SARS-CoV-2 variant landscape in NYS during December 2020–April 2021, specifically the interplay between co-circulating B.1.526 and B.1.1.7 lineages. Our findings elucidate the dynamics of competing SARS-CoV-2 variants at a time when the highly transmissible VOC Delta had overtaken B.1.1.7 worldwide and future variant displacements were likely to occur.

## Methods

### Sample Acquisition and RNA Extraction

This study was approved by the NYSDOH Institutional Review Board, under study numbers 02-054 and 07-022. The NYSDOH Wadsworth Center coordinated with >30 clinical laboratories throughout NYS that routinely submitted respiratory swabs positive for SARS-CoV-2 for whole-genome sequencing (WGS). Specimens were required to have a real-time cycle threshold value <30. We performed nucleic acid extraction on a MagNAPure 96 with the Viral NA Small Volume Kit (Roche, https://www.roche.com) with 100 μL sample input and 100 μL eluate.

### Sequencing and Bioinformatics Processing

We processed extracted RNA for WGS with a modified ARTIC V3 protocol (https://artic.network/ncov-2019) in the Applied Genomics Technology Core at the Wadsworth Center as previously described (*11*) (Appendix). We processed Illumina libraries with the ARTIC nextflow pipeline (https://github.com/connor-lab/ncov2019-artic-nf) as previously described (*14*) (Appendix).

### Sample Inclusion Criteria

We included specimens with collection dates during December 2020–April 2021 with ZIP codes of patient addresses. We removed specimens that were prescreened for specific mutations or for clinical or epidemiologic criteria. For persons with multiple specimens collected, we included only the earliest specimen.

### COVID Incidence Calculation

We obtained monthly COVID-19 case counts by ZIP code from online NYC COVID-19 data (https://github.com/nychealth/coronavirus-data) and from the NYSDOH Communicable Disease Electronic Surveillance System. We included reports with case status of confirmed or probable in the case count and assigned a month on the basis of diagnosis date. We converted ZIP code data to ZIP code tabulation area (ZCTA) and calculated incidence using population data from the 2019 1-year American Community Survey estimates (https://data.census.gov/cedsci).

### Retrospective Multinomial Space-Time Scan Statistic

We used the retrospective multinomial space-time scan statistic in SaTScan version 9.6 and applied the nonordinal method (*12*,*13*). We calculated estimated SARS-CoV-2 variant data for each ZCTA-month aggregation by multiplying the proportion of either B.1.1.7, B.1.526, or other variants in our sample by the total number of COVID-19 cases.

We set the maximum cluster size parameter a priori to 10% of the population at risk (*14*) . Space-time cluster detection in SaTScan has a noted limitation where the size of clusters cannot change over time (*15*,*16*). Given that our data are aggregated to the temporal unit of months (December 2020–April 2021), we set the maximum temporal cluster size parameter to 1 month, to enable clusters to change their shape from month to month by being designated as new clusters (Appendix).

### Inverse-Distance Weighted Interpolation and Spatial Average of SARS-CoV-2 Whole-Genome Sequencing

We used inverse-distance weighted (IDW) interpolation to visualize the spatiotemporal variation in the proportion of COVID-19 cases attributable to each SARS-CoV-2 variant in NYS and to provide estimates for these proportions in areas where we were missing data (*17*). We assigned the percentage of COVID-19 cases attributable to each variant per ZCTA to the ZCTA’s centroid for the IDW calculation. IDW interpolation generated a continuous surface of values representing the percentage of total COVID-19 cases attributed to B.1.1.7 and B.1.526, which we then averaged over each ZCTA geometry.

We then multiplied the estimated percentage of each SARS-CoV-2 variant generated from IDW interpolation by the total number of COVID-19 cases for each ZCTA and month to estimate the total number of COVID-19 cases attributable to each variant. Estimated numbers of variant cases generated geographic mean centers for each month of the study period (*18*) (Appendix).

### Phylogeographic Analyses

We incorporated into the analysis all NYS B.1.526 genomes generated by Wadsworth from the study period, barring a small fraction that did not pass quality control and those removed as redundant (Appendix). We downloaded all B.1.526 genomes from the United States and associated metadata (excluding NYS sequences) from GISAID (https://www.gisaid.org) and randomly subsampled them proportionally to their overall frequency in the United States. The final dataset included B.1.526 genomes from domestic locations (Massachusetts, New Jersey, Pennsylvania, Connecticut, California, Florida, Maryland, Michigan, Minnesota, and North Carolina), the 5 boroughs of NYC (Bronx, Brooklyn, Queens, Staten Island, and Manhattan), Long Island, the Hudson Valley and upstate New York (Western NYS, the Finger Lakes, the Capital District, and Central NYS regions). We aligned genomes in mafft 7.475 (*19*), masking problematic sites (https://github.com/W-L/ProblematicSites_SARS-CoV2). We generated a maximum-likelihood phylogeny in IQTree 1.6.12 (*20*) with 1,000 ultrafast bootstrap replicates (*21*) and time-calibrated it in TreeTime 0.7.6 (*22*). This tree served as the fixed tree for ancestral state reconstruction in Beast 2.6.2 (*23*) to infer timing and source of B.1.526 introductions within NYS. We allowed the Bayesian analysis to run for >4 million generations and monitored it in Tracer 1.7.1 (*24*) until the effective sample size of all parameters >200 and the Markov chain Monte Carlo appeared to reach stationarity.

We conducted a B.1.1.7 phylogeographic analysis in the same manner with the states inferred for a fixed topology until all effective sample sizes reached >200. The final dataset included B.1.1.7 genomes from domestic locations (Massachusetts, New Jersey, Pennsylvania, Connecticut, California, and Florida) NYC, Long Island, Mid-Hudson, Finger Lakes, southwestern NYS (the Southern Tier and western regions of NYS) and Northern NYS (Capital District, Mohawk Valley, Central NYS, and the North Country).

We generated maximum clade credibility trees for B.1.526 and B.1.1.7 in TreeAnnotator 2.6.2 (*23*) with a 10% burn-in. We summarized the number of introductions between locations by using Baltic (https://github.com/evogytis/baltic), adopting the exploded tree script for Python 3. We considered only introductions with a posterior probability >0.7. We visualized and annotated trees in FigTree 1.5.5 (http://tree.bio.ed.ac.uk/software/figtree) and ggtree (*25*) for R 4.1.0 (http://www.R-project.org) (Appendix).

## Results

### Summary Statistics

We included in the study a total of 8,517 SARS-CoV-2 specimens sequenced by Wadsworth with collection dates during December 2020–April 2021. Among the included specimens, B.1.1.7 constituted 1,107 (13%) and B.1.526 constituted 904 (10.6%) of the samples.

The earliest B.1.1.7 samples sequenced by Wadsworth were collected on December 24, 2020, from a resident of Manhattan (Metro or NYC region) and a person in Saratoga County (Capital Region). B.1.1.7 remained relatively rare among all samples through the end of January. The Metro and Capital regions experienced the earliest increases in B.1.1.7, although the proportion of B.1.1.7 did not exceed 15% through February. The proportion of B.1.1.7 increased in March across all regions, most notably in the western region, where it constituted ≈75% of all samples by the end of March and continued to rise through April. The Metro Region experienced the most gradual increase in B.1.1.7; the proportion did not exceed 40% until the end of April.

The earliest B.1.526 sample sequenced by Wadsworth was collected on December 9, 2020, from a patient in the Bronx (Metro or NYC region). The proportion of B.1.526 increased in the Metro Region throughout December, reaching 10% of total samples by the end of the month. The proportion of B.1.526 in the Metro Region approached 40% by the end of January, peaked at ≈60% in mid-February to early March, and then plateaued at ≈50% through April. B.1.526 was not consistently detected in the other regions until February and its proportion generally remained <40%. The combined proportion of all lineages other than B.1.1.7 and B.1.526 dropped to <20% in all NYS regions by the end of April.

### Cartographic Visualization

Maps of interpolated proportion of B.1.1.7 relative to all other lineages by ZCTA (Appendix Figure 1, panel A) show a general trend of spread through the southern portion of NYS in January, statewide distribution by February, diffuse increase in proportion in March, and a sustained high proportion throughout the state in April, with strong dominance in the western region. In contrast, maps of interpolated proportion of B.1.526 show more constricted initial spread focused around NYC and surrounding areas in January; statewide distribution was not achieved until March, and a moderate proportion was sustained mostly within the Metro Region (Appendix Figure 1, panel B).

**Figure 1:**
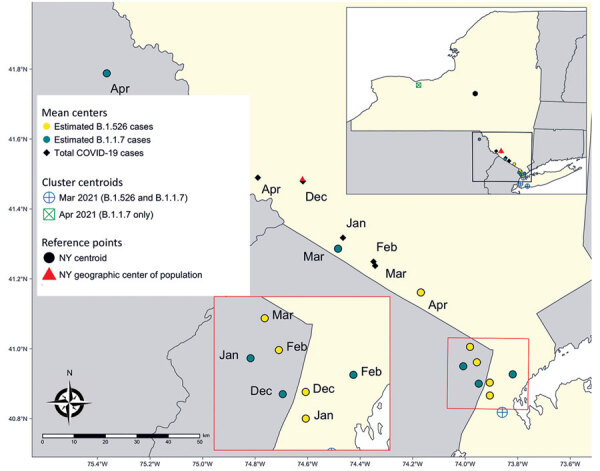
Geographically weighted mean centers of total and estimated coronavirus disease cases attributable to B.1.526 and B.1.1.7 variants, New York State, USA, December 2020–April 2021. Cluster centroids refer to the results of the multinomial space-time scan analysis (Figure 2). New York’s centroid and geographic center of population are added as reference points.

Maps of geographic mean centers of estimated B.1.1.7 and B.1.526 cases (Figure 1) show that shifts in the SARS-CoV-2 variant landscape affected the spatial distribution of COVID-19 cases overall. In December 2020, the mean center of total COVID-19 cases and the mean center of the population of NYS were nearly spatially coincident, implying that COVID-19 cases were distributed in accordance with NYS’s population. At the same time, the mean center of B.1.526 cases occurred near the NYC area, then gradually moved slightly northwest as B.1.526 expanded modestly into upstate regions. Similarly, the mean center of B.1.1.7 cases was located near NYC in December 2020, then moved northwest during March and April to a much greater degree than for B.1.526 cases, probably because of the B.1.1.7 cluster occurring in the Finger Lakes region. Consequently, the spread of B.1.1.7 in upstate NYS, especially within the western region, resulted in a northwesterly shift of the mean center of total COVID-19 cases by April 2021. The spatial shift pushed the mean center of COVID-19 cases northwest of NYS’s population center, indicating that the April B.1.1.7 cluster had an outsized effect on the overall distribution of COVID-19 cases.

### Retrospective Multinomial Space-Time Scan Statistic

**Figure 2:**
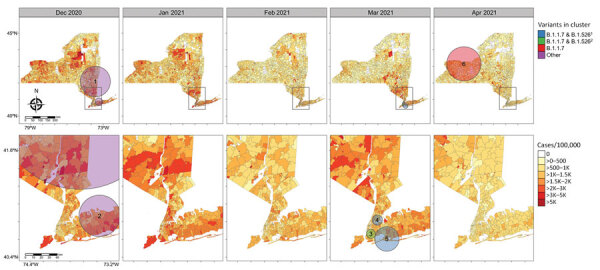
Severe acute respiratory syndrome coronavirus 2 variant clusters identified from retrospective multinomial space-time scan analysis and coronavirus disease incidence by ZIP code tabulation area, New York State, USA, December 2020–April 2021. Circles indicate clusters with relative risk >1. 1, variant includes B.1.526, B.1.526.1, and B.1.526.2; 2, variant includes B.1.526 and B.1.526.2.

Retrospective multinomial space-time scan analysis indicated 6 statistically significant clusters with elevated relative risk (RR) of COVID-19 attributable to specific variants (Figure 2; Appendix Table 1). Two clusters of elevated RR of other lineages were found in December, 2020 in the Metro and Capital regions as well as Long Island, reflecting the nearly nonexistent risk for B.1.1.7 and B.1.526 infection. Three clusters of elevated RR of multiple combinations of B.1.1.7, B.1.526, B.1.526.1, and B.1.526.2 were found in March 2021 in the NYC and Long Island regions. The sixth cluster exhibited an elevated RR of >7.0 for B.1.1.7, with a radius of 114.38 km centered in the Finger Lakes area (western and central regions) during April. In addition, the presence of B.1.1.7 and B.1.526 clusters in March and April coincide with a general statewide decrease in incidence of COVID-19 (Figure 2).

### Phylogeographic Analyses

**Figure 3:**
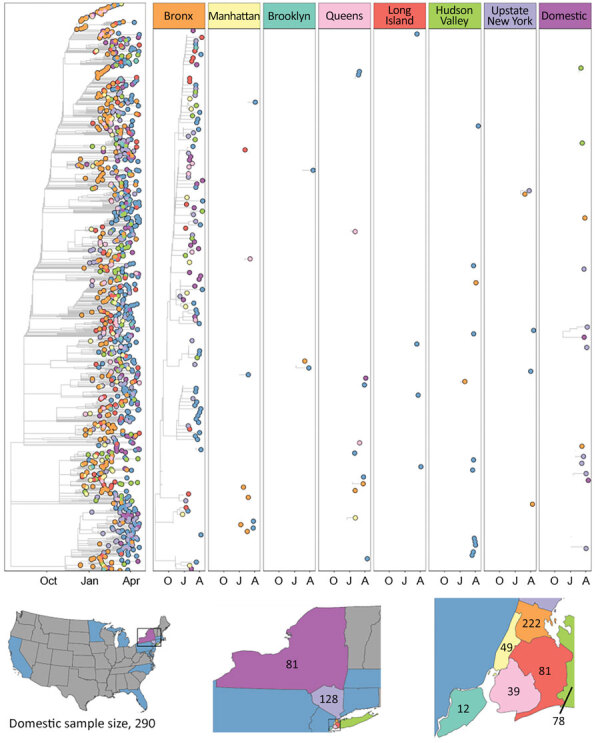
Time-calibrated phylogeny of severe acute respiratory syndrome coronavirus 2 variant B.1.526, New York and other states, USA, December 2020–April 2021. Left panel represents a maximum-likelihood phylogeny of 980 genomes from New York and other US states generated in IQTree 1.6.12 (*20*) with timescale inferred by TreeTime 0.7.6 (*22*) and ancestral state reconstruction performed in BEAST 2.6.2 (*23*). Faceted panels indicate the source of B.1.526 introductions into different regions of New York and other states (domestic). Only introductions supported by an ancestral state probability of >0.7 are shown. Bottom panel shows locations sampled and sample sizes. A, April; J, January; O, October.

The final B.1.526 dataset for phylogenetic reconstruction contained 980 genomes from all regions of NYS and various domestic locations (Bronx, 222; Hudson Valley, 128; Brooklyn, 39; Long Island, 78; Manhattan, 49; Queens, 81; Staten Island, 12; upstate NYS, 81; domestic, 290). The final B.1.1.7 dataset contained 1,195 genomes from the NYC region (181), Finger Lakes (239), Hudson Valley (78), Long Island (130), Western NYS and the Southern Tier (southwestern NYS, 56), Capital District, Mohawk Valley, Central NYS, and the north country (Northern NYS, 149), as well as other states (domestic, 362). Results from the phylogeographic analysis indicated that B.1.526 emerged within the NYC area near the end of 2020 and that the Bronx was a major source of spread to other regions of NYS and the United States (domestic) (Figure 3). Although sampling biases could have influenced the number of introductions assigned to the Bronx, the domestic category had greater representation in the dataset but led to substantially fewer introductions (Appendix Table 2). Domestic genomes represented 29.5% of the dataset but this location was responsible for only 6.7% of all B.1.526 introductions, whereas the Bronx represented 22.7% of the dataset and led to 63.8% of all introductions (Appendix Table 2). Excluding the Bronx, B.1.526 transmission between boroughs and from these boroughs to other locations was relatively infrequent. We used subsampling strategies to investigate the strength of our results from the full B.1.526 phylogeographic analysis. These strategies included evenly sampling each region or borough, sampling evenly across time (except for December, which had very few B.1.526 cases compared with other months), sampling proportionally to the total incidence of SARS-CoV-2 per region or borough, and downsampling high-incidence regions or boroughs to the mean of B.1.526 cases per month. We performed each subsampling analysis in triplicate. Despite the different subsampling strategies, the root of the tree consistently fell within NYC, and the Bronx continued to serve as a major source of B.1.526 (data not shown).

**Figure 4:**
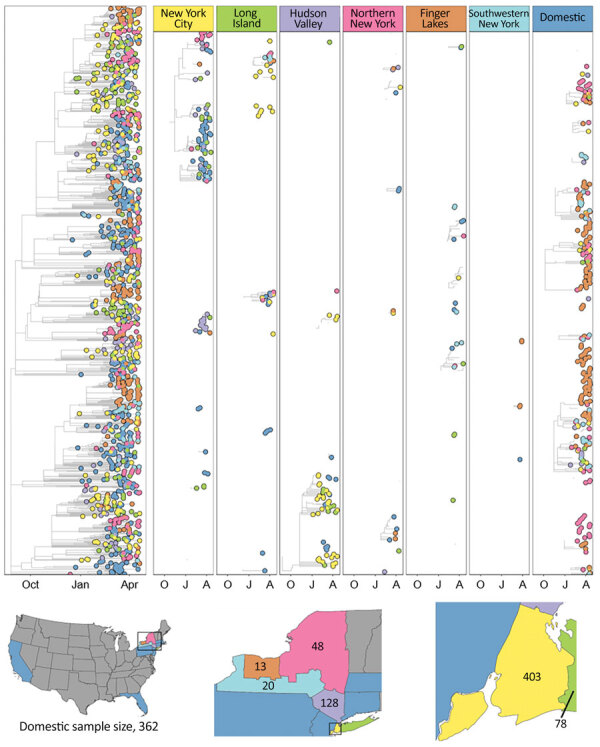
Time-calibrated phylogeny of severe acute respiratory syndrome coronavirus 2 variant B.1.1.7, New York and other states, USA, December 2020–April 2021. Left panel represents a maximum-likelihood phylogeny of 1,195 genomes from New York and other states generated in IQTree 1.6.12 (*20*) with timescale inferred by TreeTime 0.7.6 (*22*) and ancestral state reconstruction performed in BEAST 2.6.2 (*23*). The tree was rooted with a P.1 genome (not shown). Faceted panels indicate the source of B.1.1.7 introductions into different regions of New York and other states (domestic). Only introductions supported by an ancestral state probability of >0.7 are shown. Bottom panel shows locations sampled and sample sizes. A, April; J, January; O, October.

Multiple domestic introductions contributed to the initial presence of B.1.1.7 in NYS (*11*) (Figure 4), with transmission occurring most frequently in the Finger Lakes and Northern NYS (Figure 4). The Finger Lakes and Northern NYS were well-represented in the dataset (32% of genomes) but contributed substantially less to the distribution of B.1.1.7 (accounting for 13% of the total number of introductions) than domestic sites, which represented 20% of the data and were responsible for the highest percentage of introductions (≈39%) (Appendix Table 3). The Finger Lakes showed the lowest proportion of sequenced cases attributable to introductions but the largest sample size in NYS, suggesting more sustained transmission of B.1.1.7 in this region.

## Discussion

The repeated emergence of novel variants of SARS-CoV-2 has largely defined the COVID-19 pandemic response in 2021. As vaccination rates, prior exposure levels, and behavioral public health measures continuously change, so too will selective pressures (*26*). Given that selective pressures likely vary across regions, it follows that the emergence and spread of SARS-CoV-2 variants are also regionally dynamic. We combined spatial statistical, phylogeographic, and cartographic visualization techniques to examine the spatiotemporal dynamics of the VOC B.1.1.7 (Alpha) and the VOI B.1.526 (Iota) in NYS during December 2020–April 2021.

The concurrent spread of B.1.1.7 and B.1.526 offers a unique opportunity to compare the dynamics of competing variants of SARS-CoV-2 within a population during a period of substantial fluctuations in statewide COVID-19 incidence and the implementation of a vaccination campaign in January 2021. Shortly after its appearance in the Bronx in late 2020, B.1.526 quickly became the most common lineage in NYC and the surrounding region. The rapid dominance of B.1.526 in NYC is corroborated by our phylogeographic results (Figure 3), which depict widespread initial transmission within the Bronx, periodic introductions to neighboring boroughs, and later introductions to the greater Metro Region and other states. The spread of B.1.526 appears to have been spatially limited by the repeated introduction and transmission of B.1.1.7 outside NYC. However, behaviors such as differences in travel rates in NYC and between NYC and other regions probably contributed to the dynamics we observed. Similarly, the regional success of either variant may depend on the seroprevalence of the population. B.1.351 (Beta) was predicted to dominate in populations with a high degree of naturally acquired immunity because of immune evasion conferred by the E484K mutation in Spike (C.L. Althaus et al., unpub. data, https://doi.org/10.1101/2021.06.10.21258468), a mutation also observed in most NYS B.1.526 genomes. The spike mutations of B.1.526 (including the E484K mutation) were shown to reduce neutralization activity of convalescent-phase plasma and several antibodies (*10*). Thus, founder effects in an area of high transmission combined with high levels of prior exposure to SARS-CoV-2 might have provided B.1.526 its initial growth advantages in the NYC area, which was also the initial epicenter of the pandemic in the United States. Regions of NYS where B.1.526 had not yet established experienced rapid dominance of B.1.1.7 during March and April. This trend is most clearly seen in the near complete displacement of all other lineages by B.1.1.7 in Western NYS (Appendix Figure 2, panel A), resulting in a large cluster of elevated RR for B.1.1.7 cases in the Finger Lakes region during April (Figure 2). This finding is consistent with the enhanced transmissibility of B.1.1.7 in comparison to non-VOCs and non-VOIs and the conclusion that B.1.1.7 will dominate in populations with lower seroprevalence (C.L. Althaus et al., unpub. data, https://doi.org/10.1101/2021.06.10.21258468), such as those outside the NYC area.

The multinomial spatial scan detected 3 unique clusters in March 2021, all with increased RR for B.1.1.7 and B.1.526. The values for RR in each NYC cluster detail a distinct pattern: clusters centered within the Bronx, Brooklyn, and Manhattan had higher RR for B.1.526, whereas the cluster centered in east Queens and Long Island had a higher RR for B.1.1.7 (Appendix Table 1). During the months after B.1.526’s initial advantage in NYC, B.1.1.7 trends toward becoming the major variant in the Metro Region. Given the elevated reproductive number of B.1.1.7 and B.1.526 in comparison to other non-VOC/VOIs lineages (*27*) and the delayed dominance of B.1.1.7 in the Bronx compared with Long Island and Queens, we hypothesize that B.1.526 was more difficult to displace than other lineages circulating at the time. Almost no difference can be observed in the average global reproductive number of B.1.526 compared with B.1.1.7, although differences exist on a country level (*27*). This finding supports the idea that B.1.526 was generally more competitive against B.1.1.7 than background lineages, but other factors, including the location examined, probably influence our results.

Similarly, maps of the geographic mean centers of the estimated number of COVID-19 cases attributable to each variant capture the rapid spread of B.1.1.7 out of NYC and the relative inability of B.1.526 to claim a foothold outside of the Metro Region. The northwesterly shift in the trajectory of overall COVID-19 cases in April indicates that the expansion of B.1.1.7, in particular clustering in western NYS, had a measurable influence on the spatial spread of COVID-19 cases overall.

 There are some limitations to our study. A degree of selection bias exists within the dataset, given that specimens were screened by cycle threshold value and were submitted by a selected group of clinical and commercial laboratories that cannot perfectly represent all COVID-19 cases in NYS. We were unable to assess the demographic and clinical representativeness of our dataset because these data were not available to us. In addition, the number of specimens sequenced varied over the space and time of the study period, which created small sample sizes within many ZCTA-months. This limitation extended to the multinomial scan statistic, which was run with estimated values for COVID-19 cases attributable to B.1.1.7 and B.1.526, giving all ZCTAs with samples equal weight. However, the spatial scan assesses data according to their proximity to each other. In this context, ZCTAs are analyzed together rather than individually, which has the potential to reduce bias. Another consequence of our limited sampling was that our data exhibited zero samples from many ZCTAs for each month, which we addressed by using IDW interpolation of the proportion of B.1.1.7 and B.1.526 sequenced samples at the ZCTA-month level to visualize general patterns of variant proportions over geography. Phylogeographic analyses were hampered by similar limitations; uneven sampling among regions and the lack of global representation in our datasets could lead to incorrect trait assignments. Smaller sample sizes for some regions might have caused an underestimation of their contributions to variant transmission in NYS, whereas larger sample sizes might have inflated the number of introductions assigned. However, we believe our results largely capture the transmission dynamics of B.1.526 and B.1.1.7 in NYS, given that larger sample sizes did not always correspond to regions with outsized contributions to the spread of either variant, and subsampling the B.1.526 dataset consistently showed NYC as the dominant source of introductions.

Our phylogeographic and spatiotemporal analyses offer a method for evaluating the competitive advantages of co-circulating SARS-CoV-2 variants. We believe the emergence of VOI B.1.526 contributed to the slower rise of VOC B.1.1.7 as the dominant lineage in NYC compared with regions devoid of B.1.526. In this way, our study describes important dynamic interactions between variants with unequal transmissibility and is potentially generalizable to interactions between any known variants, including the highly transmissible Delta and Omicron variants and other variants to come.

Appendix Additional information about competitive advantage of SARS-CoV-2 variant of concern over variant of interest as indicated by spatiotemporal analyses, New York State, USA.
