# Supplementary material for: Spatiotemporal Analyses of 2 Co-Circulating SARS-CoV-2 Variants, New York State, USA
Source: Emerg Infect Dis. 2022 Mar;28(3):650–9. doi: 10.3201/eid2803.211972 (PMC8888210; doi:10.3201/eid2803.211972)
Supplement: Appendix — Additional information about competitive advantage of SARS-CoV-2 variant of concern over variant of interest as indicated by spatiotemporal analyses, New York State, USA. [file 21-1972-Techapp-s1.pdf]

Article DOI: https://doi.org/10.3201/eid2803.211972

# Spatiotemporal Analyses of 2 Co-Circulating SARS-CoV-2 Variants, New York State, USA

## Appendix

### Geographic Mean Center Calculation

The geographic mean center of total cases and estimated variant cases of COVID-19 were calculated using the following equation.

$$\frac{\sum_i^N x_i z_i}{\sum_i^N z_i}, \frac{\sum_i^N y_i z_i}{\sum_i^N z_i}$$

Where $x_i$ and $y_i$ denote latitude and longitude values of a ZCTA centroid, respectively, and zi denotes the number of cases recorded or estimated for a ZCTA. Centroid calculation, spatial averaging, IDW methods and maps were performed using the 'sf', 'raster', 'gstat', and 'tmap' packages in RStudio version 4.0.2, respectively (*1*–*5*).

### Retrospective Multinomial Space-Time Scan Statistic

The procedure for the multinomial scan statistic implemented in SaTScan from (*6*) is described below, using terms that apply to our research questions. The multinomial scan statistic assesses the null hypothesis of no clustering by globally testing whether the probability of acquiring a specific variant of SARS-CoV-2 relative to all variants of SARS-CoV-2 is the same in all parts of the study area. The rejection of the global null hypothesis permits for the scanning of a specific region and regions while testing the same null hypothesis locally. Specifically, the space-time scan procedure operates by searching for clusters in a "moving cylinder" fashion, such that the base of the cylinder is the spatial scan, while the height of the cylinder indicates the temporal scan. As the cylinder moves throughout the spatiotemporal study region, the test statistic is calculated for each scanning window, and the window that maximizes the likelihood ratio test statistic is selected as the most likely cluster. For specific details on the likelihood function and test statistic (*6*).

The moving cylinder method employed by SaTScan presents a key limitation for use examining disease outbreaks. The geometry of a cylinder does not allow for the change in the spatial extent of a cluster throughout time, as would be expected for a disease cluster that is spreading (*7*). Methodologies have been proposed to alleviate this problem, including the "square pyramid" method and the "flexible space-time scan statistic" (*7*,*8*). Neither the square pyramid nor the flexible space-time scan statistics were available in the SaTScan software, thus, we elected to reduce our maximum temporal cluster size to be equivalent to our time precision. Additionally, adjusting the population at risk parameter when using the multinomial scan statistic sets an upper bound for the size of a cluster according to the number of cases it will include, rather than the population at risk. In this way, clusters resulting from our analysis will not include more than 10% of the total cases during our specific time aggregation units of one month.

**Illumina Library Preparation and Sequencing**

Extracted RNA was processed for whole genome sequencing with a modified ARTIC protocol (artic.network/ncov-2019) in the Applied Genomics Technology Core at the Wadsworth Center. Briefly, cDNA was synthesized with SuperScript™ IV reverse transcriptase (Invitrogen, Carlsbad, CA, USA) and random hexamers. Amplicons were generated by pooled PCR with two premixed ARTIC V3 primer tools (Integrated DNA Technologies, Coralville, IA, USA). Additional primers to supplement those showing poor amplification efficiency (github.com/artic-network/artic-ncov2019/tree/master/primer_schemes/nCoV2019) were added separately to the pooled stocks. PCR conditions were 98°C for 30 seconds, 24 cycles of 98°C for 15 seconds/63°C for 5 minutes, and a final 65°C extension for 5 minutes. Amplicons from pool 1 and pool 2 reactions were combined and purified by AMPure XP beads (Beckman Coulter, Brea, CA, USA) with a 1X bead-to-sample ratio and eluted in 10mM Tris-HCl (pH 8.0). The amplicons were quantified using Quant-IT™ dsDNA Assay Kit on an ARVO™ X3 Multimode Plate Reader (Perkin Elmer, Waltham, MA, USA). Illumina sequencing libraries were generated using the Nextera DNA Flex Library Prep Kit with Illumina Index Adaptors and sequencing on a MiSeq instrument (Illumina, San Diego, CA, USA).

**Bioinformatics Processing**

Illumina libraries were processed with ARTIC nextflow pipelines (github.com/connor-lab/ncov2019/articnf/tree/illumine, last updated April 2020) as previusly described (*9*). Reads

were trimmed with TrimGalore (github.com/FelixKrueger/TrimGalore) and aligned to the reference assembly MN908947.3 (Wuhan-1) by BWA (*10*). Primers were trimmed with iVar (*11*) and variants were called with samtools mpileup function (*12*), the output of which was used by iVar to generate consensus sequences. Positions were required to be covered by a minimum depth of 50 reads and variants were required to be present at a frequency ≥0.75.

Lineages were determined by GISAID using Pangolin software 29, last updated May 27, 2021 (*13*). At the time of this analysis, B.1.526 was divided into a B.1.526 parent lineage and sublineages B.1.526.1, B.1.526.2, and B.1.526.3, which we analyzed separately in the multinomial scan analysis. Pangolin has since collapsed the sublineages and reassigned all to B.1.526.

**Phylogeographic Analyses**

All B.1.526 genomes from the United States (US) and associated metadata (excluding NY sequences) were downloaded from GISAID (GISAID.org) and randomly subsampled, with the number of genomes from each state sampled proportionally to their overall frequency in the US. Genomes were aligned in mafft v7.475 (*14*) with problematic sites masked according to (https://github.com/W-L/ProblematicSites_SARS-CoV2). Putative transmission clusters were identified by TreeCluster v1.0.3 (*15*) with a threshold free approach and only one representative genome was selected from each cluster if 1) all genomes derived from the same state within a one week time period or 2) all genomes derived from the same NY county within a one week time period to reduce the size of the dataset. After generating an initial ML tree in IQTree v1.6.12 (*16*) under a GTR+G substitution model, it became apparent that most states contributed minimally or not at all to the number of B.1.526 introductions into NY. It also appeared that most B.1.526 viral circulation occurred between NY and geographically proximal states (M.E. Petrone et al., unpub. data, https://doi.org/10.1101/2021.07.01.21259859). As the focus of our paper was mainly to document the spread of B.1.526 within NY as compared to B.1.1.7, we further reduced our dataset to include only states with the greatest number of sequenced B.1.526 cases and neighboring states to NY. Temporal signal was confirmed by TempEst v1.5.3 (*17*) and genomes with residuals > 0.005 were removed. The final dataset included B.1.526 genomes from MA, NJ, PA, CT, CA, FL, MD, MI, MN, and NC, aggregated as "Domestic". Because B.1.526 likely originated within the Metro region (Appendix Figure 2, panel B), we elected to keep the five boroughs of NYC (Bronx, Brooklyn, Queens, Staten Island, Manhattan) as well as Long

Island and Hudson Valley as distinct to infer the geographic origin of B.1.526 and determine transmission dynamics in this epicenter. The other regions of NY had either no or a considerably lower number of sequenced cases of B.1.526, which is consistent with the incidence of the variant in those regions. Thus, Western NY, the Finger Lakes, the Capital District, and Central NY regions were aggregated as “Upstate”. A second ML tree was generated for this reduced dataset in IQTree under a GTR +G4 substitution model with 1000 ultrafast bootstrap replicates (*18*). This tree was then input into TreeTime v0.7.6 (*19*) to estimate a molecular clock (inferred as ~4.0E-04 substitutions per site per year) and re-root the tree with the least-squares method. The time-calibrated tree was input as the fixed tree for discrete ancestral state reconstruction (a method previously validated by Alpert et al., 2021([9]) in BEAST2 v2.6.2 (*20*,*21*), using a symmetric substitution model and strict clock. All tree priors were removed from the final XML document output by BEAUTI and the BSSVS operator was turned on before running in BEAST. The Bayesian analysis was allowed to run for > 4 million generations and monitored in Tracer until the effective sample size of all parameters >= 200 and the MCMC chain appeared to reach stationarity.

A B.1.1.7 phylogeographic analysis was conducted in the same manner with the following exceptions: the tree was initially rooted with a P.1 (Gamma) representative as B.1.1.7 cases in NY had multiple origins, the five boroughs of NYC were included as the same region as it has been established that B.1.1.7 was introduced several times from non-NYC locations, the Capital District, Mohawk Valley, Central NY, and the North Country were aggregated as “Northern NY” given their proximity to each other, Western NY and its neighboring region, the Southern Tier, were grouped together as “Southwestern NY”, the Finger Lakes, the Hudson Valley, and Long Island remained distinct. B.1.1.7 locations required different coding than B.1.526 due to the substantial differences in sample sizes. For example, genomes from the Finger Lakes accounted for over 25% of the B.1.1.7 data but less than 2% of the data for B.1.526. NY regions were sampled proportionally to their contribution to the total number of B.1.1.7 cases for the state. MA, PA, CT, NJ, CA, and FL were grouped together as “Domestic” sources of B.1.1.7. Ancestral states were inferred for a fixed topology over 6 million generations in BEAST2 until all ESS reach >= 200. Maximum clade credibility trees for B.1.526 and B.1.1.7 were generated in TreeAnnotator v.2.6.2 (*20*) with a 10% burn-in. The number of introductions between locations was summarized by Baltic (https://github.com/evogytis/baltic) by adopting the

exploded tree script for Python 3. Only introductions with a posterior probability of 0.7 >= were considered. Trees were visualized in FigTree v1.4.4 (http://tree.bio.ed.ac.uk/software/figtree/) and ggtree (*22*) for R v4.1.0 (http://www.R-project.org).

**References**


1. Hijmans RJ. raster: geographic data analysis and modeling. 2020 [cited 2021 Dec 9]. https://CRAN.R-project.org/package=raster

2. Pebesma E. Multivariable geostatistics in S: the gstat package. Comput Geosci. 2004;30:683–91. https://doi.org/10.1016/j.cageo.2004.03.012

3. Pebesma E. Simple features for R: standardized support for spatial vector data. 2018 [cited 2021 Dec 9]. https://journal.r-project.org/archive/2018/RJ-2018-009/RJ-2018-009.pdf

4. RStudio Team. RStudio: integrated development environment for R. RStudio. 2018 [cited 2021 Dec 9]. https://www.rstudio.com/categories/integrated-development-environment

5. Tennekes M. tmap: thematic maps in R. J Stat Softw. 2018:84 [cited 2021 Dec 9]. https://www.jstatsoft.org/article/view/v084i06

6. Jung I, Kulldorff M, Richard OJ. A spatial scan statistic for multinomial data. Stat Med. 2010;29:1910–8. PubMed https://doi.org/10.1002/sim.3951

7. Takahashi K, Kulldorff M, Tango T, Yih K. A flexibly shaped space-time scan statistic for disease outbreak detection and monitoring. Int J Health Geogr. 2008;7:14. PubMed https://doi.org/10.1186/1476-072X-7-14

8. Iyengar VS. Space-time clusters with flexible shapes. MMWR Suppl. 2005;54:71–6. PubMed

9. Alpert T, Brito AF, Lasek-Nesselquist E, Rothman J, Valesano AL, MacKay MJ, et al. Early introductions and transmission of SARS-CoV-2 variant B.1.1.7 in the United States. Cell. 2021;184:2595–2604.e13. PubMed https://doi.org/10.1016/j.cell.2021.03.061

10. Li H, Durbin R. Fast and accurate long-read alignment with Burrows-Wheeler transform. Bioinformatics. 2010;26:589–95. PubMed https://doi.org/10.1093/bioinformatics/btp698

11. Grubaugh ND, Gangavarapu K, Quick J, Matteson NL, De Jesus JG, Main BJ, et al. An amplicon-based sequencing framework for accurately measuring intrahost virus diversity using PrimalSeq and iVar. Genome Biol. 2019;20:8. PubMed https://doi.org/10.1186/s13059-018-1618-7

12. Li H, Handsaker B, Wysoker A, Fennell T, Ruan J, Homer N, et al.; 1000 Genome Project Data Processing Subgroup. The Sequence Alignment/Map format and SAMtools. Bioinformatics. 2009;25:2078–9. PubMed https://doi.org/10.1093/bioinformatics/btp352

13. Rambaut A, Loman N, Pybus O, Barclay W, Barrett J, Carabelli A, et al. Preliminary genomic characterisation of an emergent SARS-CoV-2 lineage in the UK defined by a novel set of spike mutations. 2020 [cited 2021 Dec 9]. https://virological.org/t/preliminary-genomic-characterisation-of-an-emergent-sars-cov-2-lineage-in-the-uk-defined-by-a-novel-set-of-spike-mutations/563

14. Katoh K, Standley DM. MAFFT multiple sequence alignment software version 7: improvements in performance and usability. Mol Biol Evol. 2013;30:772–80. PubMed https://doi.org/10.1093/molbev/mst010

15. Balaban M, Moshiri N, Mai U, Jia X, Mirarab S. TreeCluster: Clustering biological sequences using phylogenetic trees. PLoS One. 2019;14:e0221068. PubMed https://doi.org/10.1371/journal.pone.0221068

16. Nguyen TH, Nguyen HL, Nguyen TY, Vu SN, Tran ND, Le TN, et al. Field evaluation of the establishment potential of wMelPop Wolbachia in Australia and Vietnam for dengue control. Parasit Vectors. 2015;8:563. PubMed https://doi.org/10.1186/s13071-015-1174-x

17. Rambaut A, Lam TT, Max Carvalho L, Pybus OG. Exploring the temporal structure of heterochronous sequences using TempEst (formerly Path-O-Gen). Virus Evol. 2016;2:vew007. PubMed https://doi.org/10.1093/ve/vew007

18. Minh BQ, Nguyen MAT, von Haeseler A. Ultrafast approximation for phylogenetic bootstrap. Mol Biol Evol. 2013;30:1188–95. PubMed https://doi.org/10.1093/molbev/mst024

19. Sagulenko P, Puller V, Neher RA. TreeTime: Maximum-likelihood phylodynamic analysis. Virus Evol. 2018;4:vex042. PubMed https://doi.org/10.1093/ve/vex042

20. Bouckaert R, Vaughan TG, Barido-Sottani J, Duchêne S, Fourment M, Gavryushkina A, et al. BEAST 2.5: An advanced software platform for Bayesian evolutionary analysis. PLOS Comput Biol. 2019;15:e1006650. PubMed https://doi.org/10.1371/journal.pcbi.1006650

21. Lemey P, Rambaut A, Drummond AJ, Suchard MA. Bayesian phylogeography finds its roots. PLOS Comput Biol. 2009;5:e1000520. PubMed https://doi.org/10.1371/journal.pcbi.1000520

22. Yu G, Smith DK, Zhu H, Guan Y, Lam TT-Y. ggtree: An r package for visualization and annotation of phylogenetic trees with their covariates and other associated data. Methods Ecol Evol. 2017;8:28–36. https://doi.org/10.1111/2041-210X.12628

**Appendix Table 1.** Multinomial cluster analysis cluster-specific relative risks*

| | | Lineage | | | | | |
|---|---|---|---|---|---|---|---|
| Cluster Number | Month | B.1.1.7 | B.1.526 | B.1.526.1 | B.1.526.2 | B.1.526.3 | Other |
| 1 | December | 0 | 0 | 0 | 0 | 0 | **1.56** |
| 2 | December | 0 | 0 | 0 | 0 | 0 | **1.56** |
| 3 | March | **2.83** | **4.11** | 0.36 | **1.48** | 0 | 0.13 |
| 4 | March | **1.32** | **2.82** | **4.44** | **3.77** | 0 | 0.18 |
| 5 | March | **4.59** | **1.66** | **1.12** | **1.71** | 0 | 0.19 |
| 6 | April | **7.49** | 0.29 | 0.27 | 0.54 | 0 | 0.11 |

*Relative risk (RR) greater than 1.0 is bolded.

**Appendix Table 2.** Number of B.1.526 introductions with posterior probability >0.7

| From | To | Introductions |
|---|---|---|
| Bronx | Domestic | 50 |
| Bronx | Hudson Valley | 24 |
| Bronx | Brooklyn | 11 |
| Bronx | Long Island | 17 |
| Bronx | Manhattan | 18 |
| Bronx | Queens | 19 |
| Bronx | Staten Island | 2 |
| Bronx | Upstate | 21 |
| Domestic | Bronx | 3 |
| Domestic | Hudson Valley | 8 |
| Domestic | Long Island | 4 |
| Domestic | Upstate | 2 |
| Hudson Valley | Bronx | 5 |
| Hudson Valley | Domestic | 15 |
| Hudson Valley | Long Island | 2 |
| Hudson Valley | Staten Island | 1 |
| Hudson Valley | Upstate | 1 |
| Brooklyn | Bronx | 2 |
| Brooklyn | Domestic | 2 |
| Brooklyn | Manhattan | 1 |
| Brooklyn | Queens | 1 |
| Long Island | Domestic | 5 |
| Long Island | Manhattan | 1 |
| Long Island | Staten Island | 1 |
| Manhattan | Bronx | 5 |
| Manhattan | Domestic | 4 |
| Manhattan | Brooklyn | 2 |
| Manhattan | Queens | 1 |
| Queens | Bronx | 2 |
| Queens | Domestic | 5 |
| Queens | Brooklyn | 4 |
| Queens | Long Island | 1 |
| Queens | Manhattan | 3 |
| Queens | Upstate | 1 |
| Staten Island | Domestic | 2 |
| Upstate | Bronx | 3 |
| Upstate | Domestic | 2 |
| Upstate | Hudson Valley | 1 |
| Upstate | Long Island | 1 |
| Upstate | Queens | 1 |

**Appendix Table 3.** Number of B.1.1.7 introductions with posterior probability >0.7

| From | To | Introductions |
|---|---|---|
| Capital | Domestic | 6 |
| Capital | Finger Lakes | 4 |
| Capital | Hudson Valley | 3 |
| Capital | NYC | 2 |
| Capital | Long Island | 1 |
| Domestic | Finger Lakes | 30 |
| Domestic | Capital | 26 |
| Domestic | NYC | 15 |
| Domestic | Southwestern | 14 |
| Domestic | Hudson Valley | 9 |
| Domestic | Long Island | 5 |
| Finger Lakes | Domestic | 7 |
| Finger Lakes | Long Island | 4 |
| Finger Lakes | Southwestern | 4 |
| Finger Lakes | Capital | 2 |
| Finger Lakes | NYC | 1 |
| Hudson Valley | NYC | 24 |
| Hudson Valley | Long Island | 7 |
| Hudson Valley | Domestic | 5 |
| Hudson Valley | Capital | 1 |
| Long Island | NYC | 11 |
| Long Island | Domestic | 8 |
| Long Island | Capital | 3 |
| Long Island | Southwestern | 2 |
| Long Island | Finger Lakes | 1 |
| Long Island | Hudson Valley | 1 |
| NYC | Domestic | 26 |
| NYC | Long Island | 12 |
| NYC | Hudson Valley | 10 |
| NYC | Capital | 4 |
| NYC | Finger Lakes | 2 |
| NYC | Southwestern | 1 |
| Southwestern | Finger Lakes | 3 |
| Southwestern | Domestic | 1 |

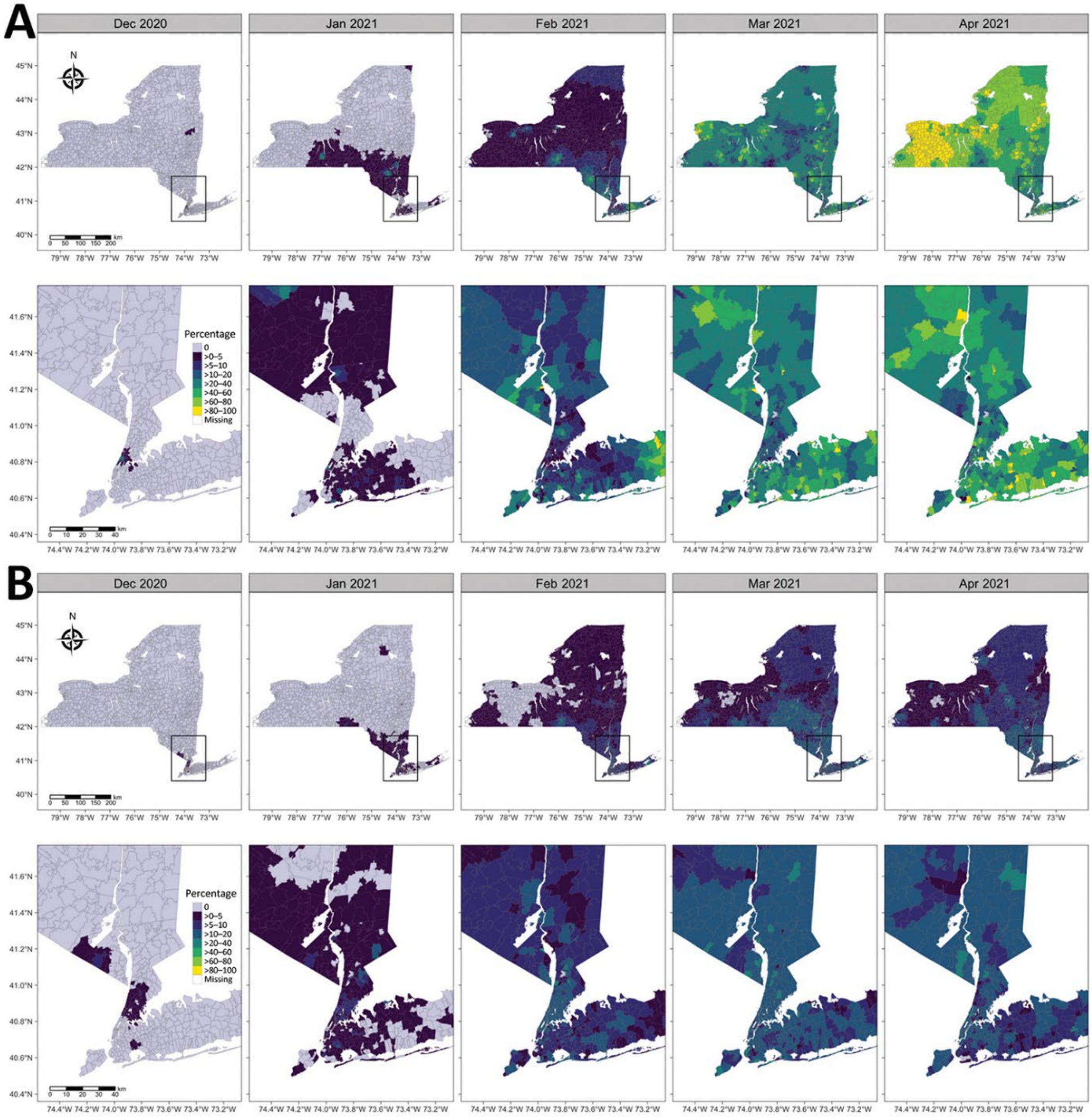


**Appendix Figure 1.** Inverse distance weighted interpolations of percentage of severe acute respiratory syndrome coronavirus 2 infections attributable to B.1.1.7 (A) and B.1.526 (B) variants compared with all other lineages, by ZIP code tabulation area, New York State, USA, December 2020–April 2021.

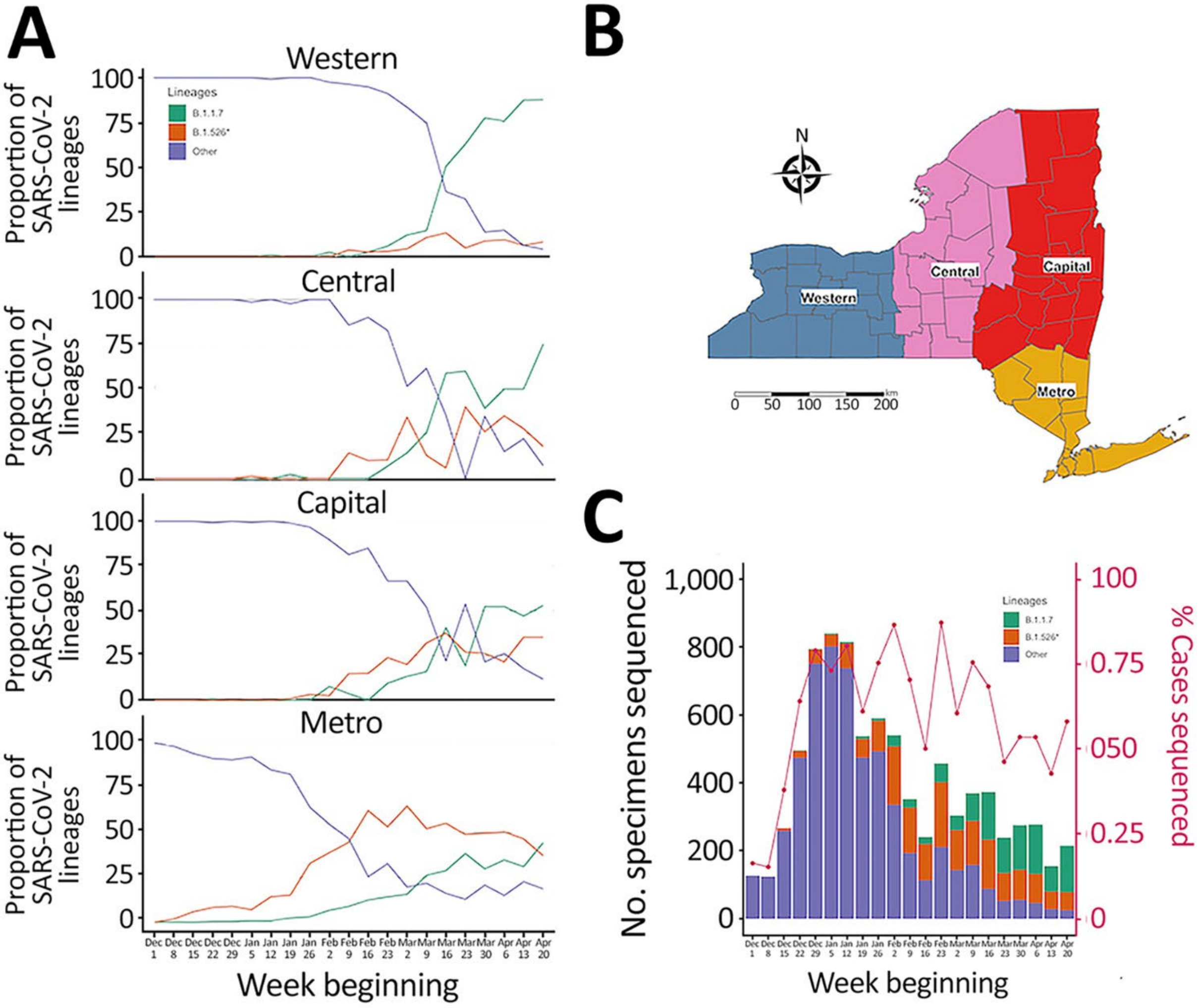
A
Western
Central
Capital
Metro
Proportion of SARS-CoV-2 lineages
Lineages
B.1.1.7
B.1.526*
Other
0
25
50
75
100
Dec 1
Dec 8
Dec 15
Dec 22
Dec 29
Jan 5
Jan 12
Jan 19
Jan 26
Feb 2
Feb 9
Feb 16
Feb 23
Mar 2
Mar 9
Mar 16
Mar 23
Mar 30
Apr 6
Apr 13
Apr 20
Week beginning
B
N
Western
Central
Capital
Metro
0 50 100 150 200
km
C
No. specimens sequenced
0
200
400
600
800
1,000
% Cases sequenced
0
0.25
050
0.75
100
Lineages
B.1.1.7
B.1.526*
Other
Week beginning
